# Book Review: Gender, Power, and Violence: Responding to Sexual and Intimate Partner Violence in Society Today

**DOI:** 10.3389/fsoc.2020.516497

**Published:** 2020-11-16

**Authors:** Karl Vachuska

**Affiliations:** Department of Sociology University of Wisconsin-Madison, Madison, WI, United States

**Keywords:** sexual violence, gender-based violence (GBV), theory, intimate partner violence (IPV), institution theory

Angela Hattery and Earl Smith's *Gender, Power, and Violence: Responding to Sexual and Intimate Partner Violence in Society Today* takes an institutional perspective in analyzing sexual violence in the United States, the mechanisms that enable it, and the potential solutions to reduce its prevalence. The authors lay out a clear theoretical orientation, identifying broad patterns of institutional characteristics that allow gender-based violence, before diving into specific institutional cases and instances that exemplify their theory. Their work provides an insightful analysis of modern institutions, such as Fraternities, the Military, and Prisons, and why and how these and other institutions continue to operate under gendered power structures and subsequently perpetuate gender-based violence utilizing a variety of past research including case studies and ethnographies. Following the #MeToo movement in recent years, and the subsequent social focus on Hollywood concerning gender-based violence, the timing of this publication in the United States is critical as it relates to the social context, addressing the phenomena of gender-based violence across a wide variety of institutions within the country. Hattery and Smith also grapple with the broader social context by highlighting the heightened levels of mass incarceration in the United States and how it interacts with the phenomena of sexual violence both directly and punitively.

The authors claim how and why institutions perpetuate violence against women, providing a broad and clear theoretical orientation. Citing Goffman's *Asylums: Essays on the Social Situation of Mental Patients and Other Inmates*, the authors emphasize the concept of the total institution and the quasi-total institution in enforcing hierarchies and controlling patterns of behavior broadly, in doing so, diving into the very dynamics that perpetuate gender-based violence within the United States in particular, such as how particular hypermasculine cultures are maintained within institutions as well as where and who maintains power over institutions. While substantial work has been done on specific institutions and how gender-based violence persists within them, this work is seminal in merging these many institutional cases which have been well-researched empirically, into a broad framework pertinent to how gender-based violence is enabled within the United States by some of the most influential American Institutions. The book should additionally be praised for presenting such an in-depth organization of modern research on sexual violence. Ultimately, the novel framework they create may be especially useful in the future examination of less-studied institutions and institutions outside of the United States.

The authors further build on their institutional framework by identifying four broad patterns of American institutional dynamics that enable gender-based violence within the United States, examining Segregated Genders, Hypermasculinity, Resistance to Integration, and Segregation in Leadership. The authors articulate how these tendencies uphold gender-based power structures within institutions and encourage toxically masculine cultures to flourish. Hattery and Smith suggest that institutions enable gender-based violence via Hypermasculinity and Power, perpetuating tendencies to behave in ways that demonstrate and confirm the men's masculinity to one another as well as perceptively and symbolically reinforcing “the inferiority of women.” The authors follow up on their initial identification and description of these patterns throughout the book by applying them to the various institutions they examine. For example, the authors highlight “Hypermasculinity” in discussing Fraternities in Chapter Three, emphasizing the role it plays in enabling and promoting men to behave toxically, “excessive drinking and identifying women with whom one can have sex” in order to conform to masculinity norms. The authors rely primarily on historical analyses and ethnographic evidence conducted by others, but include some of their empirical research and include analysis of highly-publicized relevant incidents/cases (such as an analysis of the Larry Nassar scandal). In all cases, they rely on a wide variety of evidence both in form and example in supporting their claims and conducting each of their institution-specific analyses.

Within each case study, the authors offer an organized and uniquely prepared answer to “What can be done?” again acknowledging the original unique patterns and conditions (such as the nature of inherent structural inequality, like sex segregation, or having the status quo for investigations to be handled internally) that make up the qualities of the institution that allows it to perpetuate sexual violence and offering nuanced solutions that address these flaws. They repeatedly emphasize broad notions of the need for offenders to be held accountable more often, to more greatly deter behavior as well as rehabilitate offenders, and eliminate chronic re-offending, emphasizing the need for institutional culture to change, offering tailored solutions for specific institutions, such as suggesting in Chapter Six that misconduct by athletes should no longer be handled internally by universities or sports organizations, as they traditionally have been in the United States.

The authors, Angela Hattery, a professor of Women and Gender Studies, and Earl Smith, a sociology and American ethnic studies professor, contribute a broad yet powerfully nuanced array of knowledge in this work. While both have studied a wide variety of sociological topics as well as specific issues related to Sexual Violence, their collective experience with the Sociology of Race, as well as Smith's experience in the Sociology of Sport lends itself to a detailed and specialized analysis within the book, such as engagements with the “Cool Pose” and structural racism's interaction with gender-based violence.

Hattery and Smith do an excellent job of keeping their analyses of specific American institutions grounded in the theory they present at the beginning of the book. They explicitly identify what type of institution they believe each case study to be, explaining their logic and offering concise evidence into how that institutional type plays a role in how that particular institution uniquely perpetuates sexual violence. They further apply their four-pattern framework to deeply examine how their institutional patterns play a specific but generalizable role in how different institutions perpetuate sexual violence distinctly.

Another excellent piece of Hattery and Smith's work is dealing with the delicate issue of how to increase the accountability of sexual violence offenders in an era of excessive mass incarceration in the United States, laying out a clear and explicit approach that delicately satisfies a seemingly unsolvable social problem. Their approach emphasizes always holding offenders accountable and always putting the survivor first in the process to minimize the further trauma/challenges they may subsequently experience. Of importance, though, Hattery and Smith suggest that incarceration should not be the solution for dealing with perpetrators of sexual violence, as it traditionally is in the United States. Instead, they emphasize the need for a rehabilitative approach, which places minimizing the offender's likelihood of committing further acts as the primary goal. While Hattery and Smith fail to fully connect their structural analyses to their solutions, there is significant merit to their proposed approaches.

One weakness of Hattery and Smith's work is that they often fail to offer enough quantitative warrants for their many general or specific claims. They instead rely almost exclusively on citing various ethnographic evidence. While most of their arguments are still convincing given the depth of the description they present, quantitative warrants would boost many of their claims, both large and small, and further portray the extent of particular phenomena. This is particularly problematic, as there seem to be many topics where statistics seem to be readily available as evidence, such as in examining Prisons, in Chapter five. Generally, overemphasis on nuanced details of specific case studies paints an unconvincing broader picture. For instance, in Hattery and Smith's description of the fraternal practice in Chapter Three, based on other's ethnographic works, the authors describe in detail a wide variety of highly-detailed fraternal practices before generalizing such practices to being characteristic of fraternities across the United States. However, such specific and nuanced fraternal practices being described as universal American practices leaves the reader questioning the validity of the authors' generalization of specific ethnographic observations and the general breadth of data they use to substantiate these ideas.

Hattery and Smith's work is ultimately useful in laying out a theoretical orientation to power and sexual violence and grounding it neatly in the United States in modern American examples and cases. While quantitative evidence is lacking, there is significant merit in the full description they present throughout. Besides delving into a robust analysis of current widely-known examples of sexual violence, the authors also offer well-thought potential solutions to reduce the high levels of sexual violence in the United States, at broad and institutionally-specific levels. *Gender, Power, and Violence: Responding to Sexual and Intimate Partner Violence in Society Today* serve as a compelling and informative work for academics and the public alike, who seek to expand their knowledge and understanding of how modern sexual violence is enabled within the United States, or seek a theoretical orientation in regards to the institutional perpetuation of gender-based violence more broadly.

## Author Contributions

The author confirms being the sole contributor of this work and has approved it for publication.

## Conflict of Interest

The author declares that the research was conducted in the absence of any commercial or financial relationships that could be construed as a potential conflict of interest.

